# PRSS1 mutation: a possible pathomechanism of pancreatic carcinogenesis and pancreatic cancer

**DOI:** 10.1186/s10020-019-0111-4

**Published:** 2019-09-14

**Authors:** Qicai Liu, Ling Guo, Sheng Zhang, Jingwen Wang, Xinhua Lin, Feng Gao

**Affiliations:** 1 Department of Reproductive Medicine Centre, 1st Affiliated Hospital, Fujian Medical University, 20 Chazhong Road, Fuzhou, 350005 China; 2 Department of Pharmaceutical Analysis, Fujian Medical University, Fuzhou, Fujian China; 3 Department of Pathology, 1st Affiliated Hospital, Fujian Medical University, Fuzhou, Fujian China; 4 Shool of Basic Medical Sciences, Fujian Medical University, Fuzhou, China

**Keywords:** Pancreatic cancer, PRSS1 mutation, JAK1-STAT5, Transgenic mouse model

## Abstract

**Background:**

Previous studies revealed somatic mutations of the cationic trypsinogen gene (PRSS1) in patients with chronic pancreatitis and pancreatic cancer. However, whether PRSS1 mutations trigger pancreatic cancer and/or promote malignant proliferation and metastasis in pancreatic cancer remains largely unclear, as well as the potential underlying mechanisms.

**Methods:**

In the present study, whole-exome sequencing was applied for screening, and the R116C mutation was validated by Sanger sequencing. Phosphorylation antibody array, RNA-Seq, and RT-qPCR were adopted to screen and validate that R116C mutation promoted pancreatic cancer progression via the JAK1-STAT5 pathway.

**Results:**

It showed that migration and invasion were significantly increased in R116C-bearing PANC-1 cells compared with wild type counterparts. In a transgenic mouse model of iZEG-PRSS1_R116C, primary pancreatic intraepithelial neoplasia (PanINs) was observed in the pancreatic duct.

**Conclusions:**

These findings suggested a novel pathway mediating pancreatic cancer development, with PRSS1 mutation and overexpression playing an “inside job” role in pancreatic carcinogenesis and tumor development.

**Supplementary information:**

**Supplementary information** accompanies this paper at 10.1186/s10020-019-0111-4.

## Introduction

Currently, there is no direct evidence supporting pancreatitis transformation into pancreatic cancer (Campa et al., 2018; Kleeff et al., 2017). However, certain genetic factors predispose the transformation of chronic pancreatitis into pancreatic cancer, and could be more precisely characterized as common factors (Campa et al., 2018; Rustgi, 2014; Lowenfels et al., 1993). Among them, the most distinguished is the cationic trypsinogen gene (PRSS1) (Rebours et al., 2008; Hezel et al., 2006), even though it is responsible only for a small portion of pancreatic cancer cases.

To date, all known PRSS1 mutations are somatic, with genetic tendency and dominant traits, which is different from EGFR/KRAS/BRAF gene mutations present in the context of cancerous tissues; somatic mutations are an important causal factor of tumorigenesis and a critical element for tumor heterogeneity (Hezel et al., 2006; Chang et al., 2017; Le Maréchal et al., 2006). Meanwhile, trypsinogen is specifically and highly expressed in the pancreas, and trypsin activates protease-activated receptor-2 (PAR-2); this results in cell cycle disturbance via the extracellular signal-regulated kinase (ERK) pathway, triggering pancreatic cancer occurrence (Jiang et al., 2010; Soreide et al., 2006). Moreover, trypsin can augment the malignant behavior of tumors, and stimulates tumor cell proliferation and invasion by degrading the extracellular matrix (ECM) upon activation (Jiang et al., 2010; Soreide et al., 2006). In this regard, PRSS1 mutation attracts increasing attention in the assessment of pancreatic carcinogenesis and cancer development.

In our previous study, the PRSS1 mutations p.T135A, p.T137 M, and p.C139S were detected in pancreatic cancer patients, with the PRSS1_rs10273639 genotype impacting the clinical outcome of this malignancy (Liu et al., 2012; Liu et al., 2008; McWilliams et al., 2009). However, well-designed studies have not addressed many underlying questions. For example, whether PRSS1 mutations detected in peripheral blood samples from pancreatic cancer patients are causal factors of pancreatic carcinogenesis is unclear. In addition, it remains unknown whether PRSS1 mutations enhance malignant proliferation and invasion in pancreatic cancer. Furthermore, the pathways adopted by these mutations to promote tumorigenesis and tumor development are largely undefined. In the present study, patient specimens as well as in vitro and in vivo assays, including a genetically engineered animal model, were applied to demonstrate that mutated trypsin triggers pancreatic carcinogenesis and the malignant behaviors of proliferation and invasion in an “inside job” mode.

## Materials and methods

### Next generation sequencing (NGS)

There were two families recruited for full-exon screening. Screening matching of family members was: 2 PDAC (pancreatic ductal adenocarcinoma) cases VS 3 healthy first-degree relatives; 1 PDAC case plus chronic pancreatitis 1 case VS 2 healthy first-degree relatives (Additional file 1). A total of 172 patients were enrolled from East China (Fujian and Zhejiang provinces), after diagnosis of PDAC by pathology or needle biopsy. This study was carried out in accordance with the recommendations of Medical ethics committee of the First Affiliated Hospital of Fujian Medical University. The protocol was approved by the Medical ethics committee of the First Affiliated Hospital of Fujian Medical University.

### Pathogenicity gene analysis

Variants, including SNPs (single nucleotide polymorphisms) and short Indels (insertions and deletions), revealed in raw data were filtered by data quality, and the remaining variants were blasted against the dbSNPl37, 1000 Genome project, HapMap Project, and pancreatic cancer databases to exclude virulence genes. The remaining variants were analyzed with the SIVF software and Polyphen 2 software to uncover the mutation loci which generate amino acid variations, and to sort out pathogenicity genes by combining the familial inheritance pattern. The potential pathogenicity genes obtained after data analysis were validated by Sanger sequencing in the patients and their family members.

### Assessment of trypsin effect on PANC-1 cell growth

They were cultured in a 37 °C incubator for 24 h, followed by addition of trypsin (100 μL/well; GIBCO, California, USA), diluted with serum-free DMEM to 0, 10, 20, 30, 40, and 50 μg/mL, respectively. After gentle shaking, the plate was incubated for another 24 h, followed by addition of CCK8 reagent (10 μL/well) (Beyotime Biotechnology, Shanghai, China) and incubation for 2 h at 37 °C, prior to absorbance measurement at 450 nm on a microplate reader (Thermo Fisher Scientific, Massachusetts, USA).

### Detection of cell proliferation by flow cytometry (FCM)

The assay was performed with the kFluor555-EdU cell proliferation kit (KGA336, KeyGEN Biotech, Nanjing, China). Briefly, the cells were seeded in 6-well plates and cultured until adherence, followed by addition of MEdu (10 μL/well) and incubation for 24 h. The next day, the cells were trypsinized and collected, followed by fixation with 4% paraformaldehyde, permeabilization with 0.1% Triton X100, staining with the fluorescence dye kFlour555, and incubation in the dark for 30 min. After centrifugation and resuspension, the cells were analyzed on a flow cytometer (FACSCanto™ II, BD, New Jersey, USA).

### Detection of protein expression by Western blotting

Cells at the log growth phase were seeded on 6-well plates (2 × 10 (Rebours et al., 2008)/well) and cultured for 48 h, followed by digestion with trypsin (GIBCO, California, USA) and harvesting, addition of RIPA lysis buffer (Beyotime Biotechnology, Shanghai, China) and protease inhibitor (Beyotime Biotechnology, Shanghai, China), incubation on ice for 30 min, and centrifugation at 4 °C and 12,000 g for 10 min. The supernatant was then collected, followed by protein quantitation by the BCA method. Equal amounts of protein (25 μg) were resolved by 10–12% SDS-PAGE and transferred onto polyvinylidene fluoride (PVDF) membranes. After blocking with 5% skim milk for 2 h at room temperature with gentle shaking, blots were incubated with primary antibodies (all from ABclonal Biotechnology Co., Ltd., China; 1:1000) (mTOR: A2445, P6170; DDIT4: A8086, P7159; MMP:A1963, P6071; P53:A5761, Q45) overnight at 4 °C. Then, the blots were rinsed three times with TBST buffer (10 min each), followed by incubation with secondary antibodies (1:1000) for 1 h. Ultra ECL chemiluminescence was used for color development and exposure. Qualitative analysis was conducted after autoradiography. The experiment was repeated 3 times. Serum trypsin was extracted for enzyme-linked immunosorbent analyses (ELISA) (Lisu, Shanghai, China).

### Analysis of cell migration and invasion

PANC-1 cells at the log growth phase were pre-starved for 24 h by culture in serum-free DMEM, detached with 0.25% trypsin (GIBCO, California, USA), and centrifuged, prior to resuspension of single cells in serum-free DMEM (Hyclone, Utah, USA). According to preliminary experiments, cell density was adjusted to 2.5 × 10 (Rebours et al., 2008)/mL and 5 × 10 (Rebours et al., 2008)/mL for migration and invasion assays, respectively; cell viability exceeding 95% was confirmed by trypan blue staining. A total of 100 μL of cell suspension was added to the upper chamber of the invasion system (Corning, USA) in triplicate, while the lower chamber was loaded with 600 μL of culture media containing 20% FBS (GIBCO, California, USA). After incubation for 48 h, the porous membrane was dismantled, and cells not penetrating through the membrane were wiped off with cotton balls. Then, the membrane was fixed with 4% paraformaldehyde for 20 min, and subsequently stained with 0.1% crystal violet for 10 min. Cells penetrating through the membrane were counted under a microscope, analyzing 5 high-power (200×) fields randomly selected.

### RNA-SEQ

Transcriptome deep sequencing (RNA-seq) was performed using total RNA isolated from LV-NC, wild-type-PRSS1 overexpressing (OE), and R116C mutant- overexpressing PANC-1 cells, as well as pancreatic cancer, chronic pancreatitis, and adjacent normal tissue specimens. RNA-seq reads were quality filtered using SolexaQA packages with default parameters, with requisite length of 470 bp for both ends in each read pair. The sequencing data have been submitted to the NCBI Sequence Read Archive. Genes that showed a significant (*P* < 0.05) difference in transcript levels were termed differentially expressed (DE) genes.

### Localization of CD4/FOXP3, CK7, ERK/VEGFR2, and trypsin/PAR2

Tregs were identified by staining with anti-CD4and anti-Foxp3 antibodies. CD4 was detected with a rabbit antihuman antibody (Sanying, Wuhan, China) and labelled with a goat anti-rabbit secondary antibody conjugated to Cy3. Foxp3 was detected with a mouse anti-human antibody (Santa, USA) and labelled with a goat anti-mouse secondary antibody conjugated to FITC. Cells were counterstained with DAPI. These tissue samples were performed from patients with pancreatic cancer or those with chronic pancreatitis who were misdiagnosed as pancreatic cancer.

EC was identified by staining with anti-CK7, and detected with a rabbit anti-human antibody (Santa, USA) and labelled with a goat anti-rabbit secondary antibody conjugated to Cy3. DAPI was used for counterstaining.

Trypsin was detected with a rabbit anti-human antibody (ABclonal, Wuhan, China) and labeled with a goat anti-rabbit secondary antibody conjugated to Cy3 (Beyotime, Shanghai, China). PAR2 was detected with a mouse anti-human antibody (Santa Cruz, CA, USA) and labeled with a goat anti-mouse secondary antibody conjugated to FITC (Beyotime, Shanghai, China). ERK was detected with a rabbit anti-human antibody (Sangon, Shanghai, China) and labeled with a goat anti-rabbit secondary antibody conjugated to Cy3. VEGFR2 was detected with a rabbit anti-human antibody (Sangon, Shanghai, China) and labeled with a goat anti-rabbit secondary antibody conjugated to FITC. Cells were counterstained with DAPI.

### Establishment and maintenance of human PRSS1 transgenic mouse models

Mouse model establishment was outsourced to Shanghai Biomodel Organism Science & Technology Development Co., Ltd. The transgenic mouse line was housed in regular conditions, at a room temperature of 22 ± 2 °C and 40–60% humidity. Mice had free access to food and distilled drinking water. The cage was changed at least twice weekly to ensure cleanness. Breeding of the transgenic line was performed as follows. Reproductive-age mice (> 8 weeks) with appropriate genotype (mtPRSS1−/−) were housed in the same cage with heterozygote mice (PRSS1-Cre), at a female to male ratio of 1:1 or 2:1. The next generation would be delivered in around 21 days after female mice became pregnant. New-born mice at day 7–10 were tailed-clipped for genotyping, and weaned at about 4 weeks old according to male/female. All methods or assays applied in this study were approved by the animal ethics committee.

Gene knock-in processes were performed as follows. The piggyBac mRNA was transcribed from a construct in vitro, and co-transduced with a vector containing the gene-of-interest into the fertilized egg of a C57BL/6 J mouse by microinjection. The transgenic founder was then expected to come to life.

### Phospho explorer antibody Array analysis

The Phospho Explorer Antibody Array (PEX100) was designed and manufactured by Full Moon BioSystems (Sunnyvale, CA, USA). Data were collected and analyzed by Wayen Biotechnologies (Shanghai, China). Cell lysates obtained from R116C and WT PANC-1 cells were biotinylated with an Antibody Array Assay Kit. The phosphorylation ratio was calculated as follows: phosphorylation ratio = phosphorylated value/non-phosphorylated value. Significantly changed phosphoproteins (*p* < 0.05) over 1.2-fold (up- or downregulation) were included. The related pathways were analyzed in the DAVID online database.

## Results

### Elevated trypsin is a risk factor for pancreatic cancer

Serum trypsin levels were significantly higher in both patients with pancreatic duct adenocarcinoma (PDAC) and pancreatitis compared with normal control values (*p* < 0.0001 and *p* < 0.0001, respectively). These findings indicated the occurrence of pancreas damage in both pancreatitis and pancreatic cancer. Therefore, serum trypsin levels in pancreatic cancer patients were lower than in subjects with pancreatitis (*p* = 0.0168). In PANC-1 cells cultured in serum-free medium, exogenous trypsin overtly enhanced cell proliferation, which was reduced after addition of a trypsin inhibitor (Additional file 2). This indicated the outstanding effect of trypsin in pancreatic cancer cell proliferation.

In chronic pancreatitis (CP), the pathological features of microenvironment and Treg cell distribution were highly consistent in both diseases, as reflected by the conspicuous increase of Treg cells (Additional file 2). In addition, histopathological results demonstrated heterogeneity in pancreatic structure and cell organization for CP and PDAC, disorganized duct epithelial cells, overt hyperplasia of small duct epithelial cells (CK7 positive cells), nuclear enlargement, and disorganized and increased karyokinesis (Additional file 2).

#### PRSS1 mutations in PDAC

**Fig. 1:**
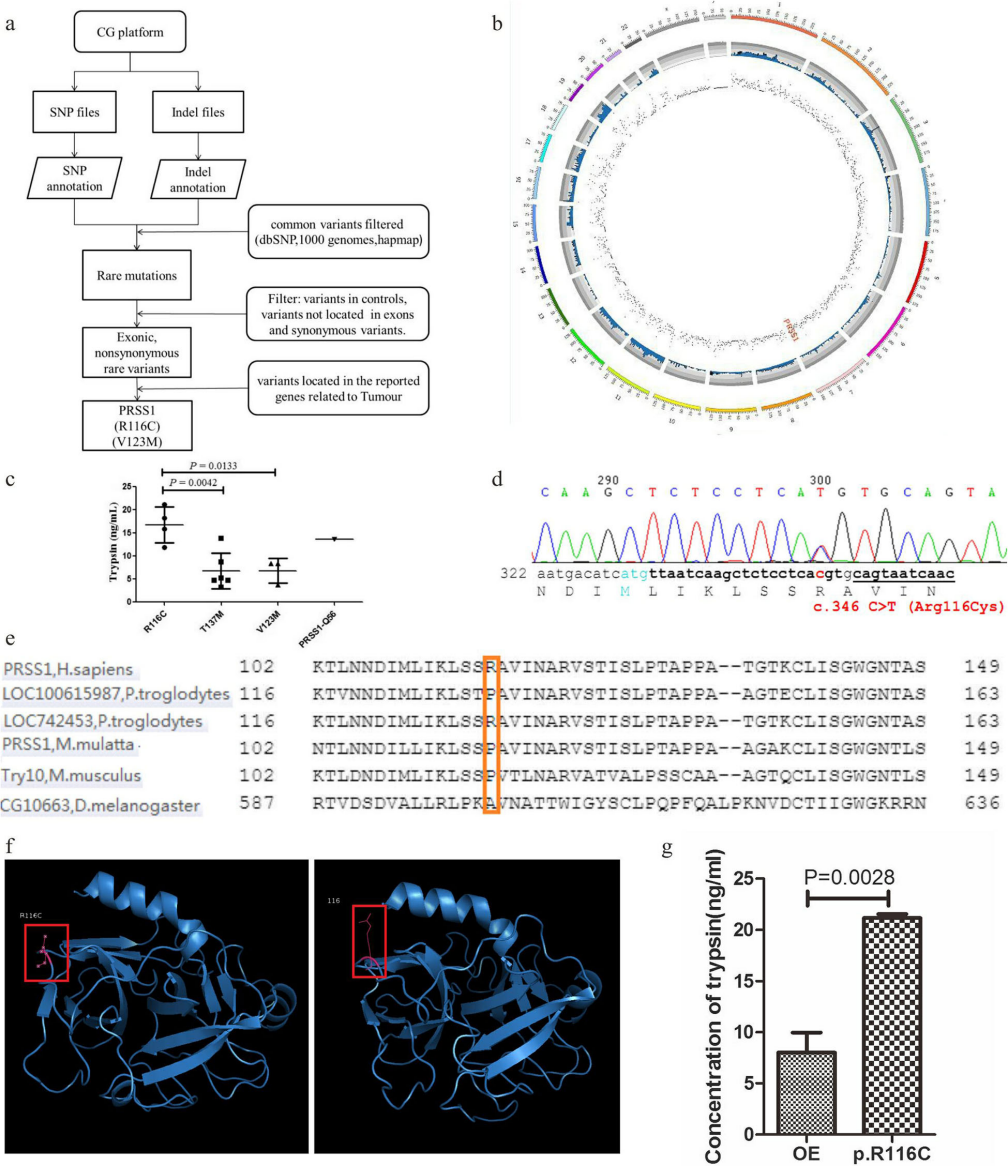
PRSS1 mutation screening and sequencing. **a**. Screening processes; **b**. Results of whole-exome sequencing screening: gray (all mutations; green (mutations following certain inheritance patterns), blue (likely detrimental mutations), red (mutations located in the PRSS1 gene sequence); **c**. PRSS1 exon mutation frequency and associations of mutations with serum trypsin levels; **d**. Sanger sequencing to confirm the R116C mutation; **e**. R116C mutation at a relatively stable site in species; **f**. PRSS1_R116C mutation could elicit protein misfolding and conformational disorder of trypsinogen; **g**. PRSS1_R116C mutation upregulated trypsin expression

After filtering out mutations in healthy individuals and sorting mutations relevant to tumor or immunology (Fig. 1a), 586 gene mutations were ultimately identified, including PRSS1 (Fig. 1b). In our previous study, PRSS1 gene mutations were detected in peripheral blood samples from pancreatic cancer patients; in addition, trypsin is a pancreas specific enzyme. In this study, PRSS1 mutation had an inheritance pattern with dominant traits. Therefore, we focused on PRSS1 for further validation. In this study, Sanger sequencing revealed that out of 172 PDAC cases, 4 harbored the R116C mutation (2.32%), and 3 had the V123 M mutation, 6 had the T137 M mutation (including one PDAC case from family b), and 1 patient had the truncating mutation Q56*; all were heterozygous mutations. Serum trypsin levels were remarkably elevated in PRSS1_R116C harboring patients (Fig. 1c); trypsin results in super-fast G1/S transition via the ERK signaling pathway, which could trigger pancreatic carcinogenesis. Moreover, trypsin can promote malignant behaviors of tumor cells. Considering the mutation rate, genetic factors, and trypsin expression levels, R116C was selected as the functional study object (Fig. 1d). By PolyPhen-2 prediction of functional effects of human nsSNPs, we found that the R116C mutation occurs at a relatively stable site in species (Fig. 1e), and could generate protein misfolding in conformational structure (Fig. 1f). In addition, trypsin levels in lysates of R116C mutation-harboring PANC-1 cells were significantly higher than those of wild type cells (Fig. 1g), consistent with clinical data.

### PRSS1_R116C augments malignant behaviors in pancreatic cancer cells

**Fig. 2:**
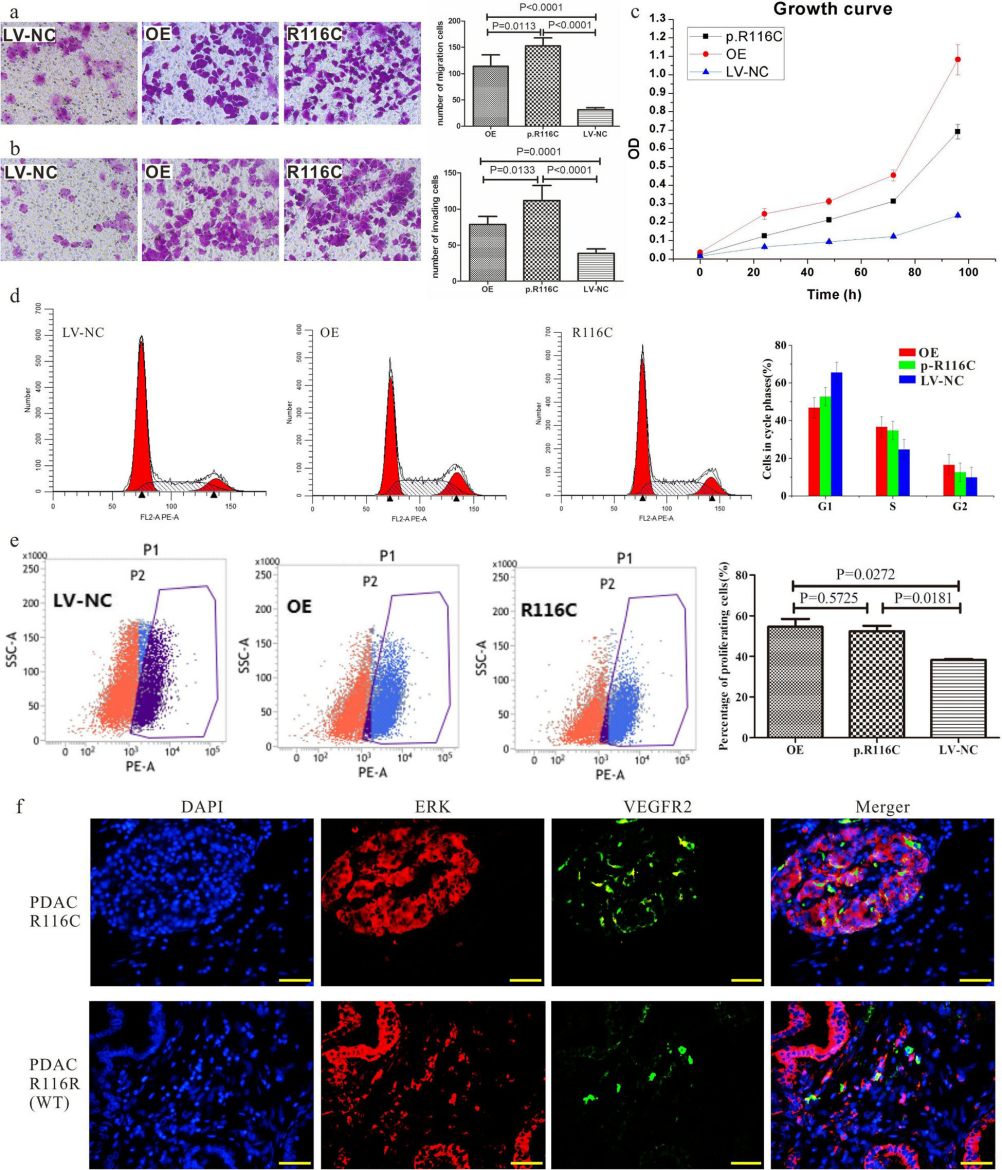
PRSS1_R116C mutation promotes the malignant phenotype of PANC-1 cells. **a**. The transwell chamber system was applied to assess the effect of PRSS1 wild type/R116C mutant overexpression on PANC-1 cell migration (converted microscope, 200×); **b**. The matrigel assay was performed to evaluate the effect of PRSS1 wild type/R116C mutant overexpression on PANC-1 cell invasion; **c**. CCK8 assay was used to measure PANC-1 cell proliferation; **d**. Effect of PRSS1 wild type/R116C mutant overexpression on PANC-1 cell cycle distribution; **e**. Flow cytometry was employed to measure the effect of PRSS1 wild type/R116C mutant on PANC-1 cell proliferation; **f**. R116C mutation prominently increased ERK and VEGFR2 expression in pancreatic cancer

To validate the differential effects of the PRSS1_R116C mutation and increased trypsin levels in promoting pancreatic cancer progression, a variety of assays and approaches were applied. Transwell migration assay showed significantly higher migration rates in PANC-1 cells harboring PRSS1_R116C (R116C) and overexpressing wild type PRSS1 (OE) compared with wild type PANC-1 cells (LV-NC) (*n* = 3, both *p* < 0.0001); this rate was significantly higher in R116C cells compared with the OE group (*n* = 3, *p* = 0.0113) (Fig. 2a). A similar trend was obtained for the invasion assay. Compared with LV-NC, the invasive capability was significantly higher in OE or R116C (*n* = 3, both *p* < 0.0001), and elevated in R116C compared with OE (n = 3, p = 0.0113) (Fig. 2b). These data indicated that trypsin could augment malignant behaviors in pancreatic cancer cells, while the R116C mutation enhanced migration and invasion in PANC-1 cells. Under the same culture conditions, the R116C group proliferated faster than LV-NC cells (Fig. 2c).

As shown previously, trypsin activates its receptor PAR-2, which results in super-fast transition of cell cycle G1/S phases via the ERK signaling pathway. In this study, the percentages of cells in the S phase were 34.69 ± 1.69% and 36.63 ± 1.97% in R116C and OE cells respectively, with no significant difference between these two groups; however, these values were higher than those of LV-NC cells (24.57 ± 5.40%). Meanwhile, G2/G1 ratio showed no difference among all groups, indicating that trypsin could cause S phase cell cycle arrest, without impacting G1/G2 phase transition in PANC-1 cells. In addition, PRSS1_R116C mutation itself did not impact the cell cycle, whereas mutation-associated trypsin overexpression triggered changes in cell cycle distribution (Fig. 2d). Flow cytometry demonstrated cell proliferation was significantly enhanced in PANC-1 cells overexpressing wild type- or R116C compared with LV-NC cells (*p* = 0.0272 and *p* = 0.0181, respectively), with no significant difference between R116C mutant and wild type transfected cells (*p* = 0.5725). Trypsin can activate the cell surface receptor PAR-2 and further activate the ERK pathway. Compared with CP tissues (harboring wild type PRSS1), ERK and VEGFR2 expression levels were obviously higher in pancreatic cancer tissues carrying the PRSS1_R116C mutation (Fig. 2e).

#### PRSS1_R116C mutation impacts the pathway mediating pancreatic carcinogenesis

Trypsin is commonly assessed as a tool enzyme for tumor studies and rarely evaluated for its promotive effects on tumor, especially pancreatic neoplasms (trypsin is specifically and highly expressed in the pancreas). Certainly, the pathway mediating its effect remains unclear. To explore the molecular mechanism in detail, we used a phospho-antibody array and screened more than 200 molecules clustered in twelve cancer-related pathways and found that highest variation in R116C cells. We found that factors or proteins showing substantial changes were enriched in JAK1-STAT5 cancer pathways. Phosphorylation antibody array and Ingenuity Pathway Analysis (IPA) were adopted for analyses (Additional file 3), and JAK1-STAT5 signaling was preliminarily identified as the pathway mediating the effect of the PRSS1_R116C mutation, belonging to phosphorylated proteins with differential expression (> 1.2 fold change) (Additional file 4). Next, RNA-seq was applied to analyze gene differential expression in cells with PRSS1_R116C (R116C) overexpression vs wild type (LV-NC), with *p* < 0.05 set as cutoff (Additional file 5). The GSEA (gene set enrichment analysis) software was applied for pathway enrichment analysis, and the results demonstrated that R116C mutation-associated differentially expressed proteins were enriched in the JAK1-STAT5 pathway (Additional file 5). Furthermore, it was demonstrated in a transgenic mouse model that R116C mutation promoted pancreatic carcinogenesis via the JAK1-STAT5 pathway (Additional file 6). Based on these findings, we proposed that the effect of the PRSS1_R116C mutation could be mediated as follows: PRSS1_R116C mutation promoted pancreatic carcinogenesis and tumor development via the canonical JAK1-STAT5 pathway, which was triggered by upregulation if trypsin, which activated its cell surface receptor PAR-2 (F2RL1).

**Fig. 3:**
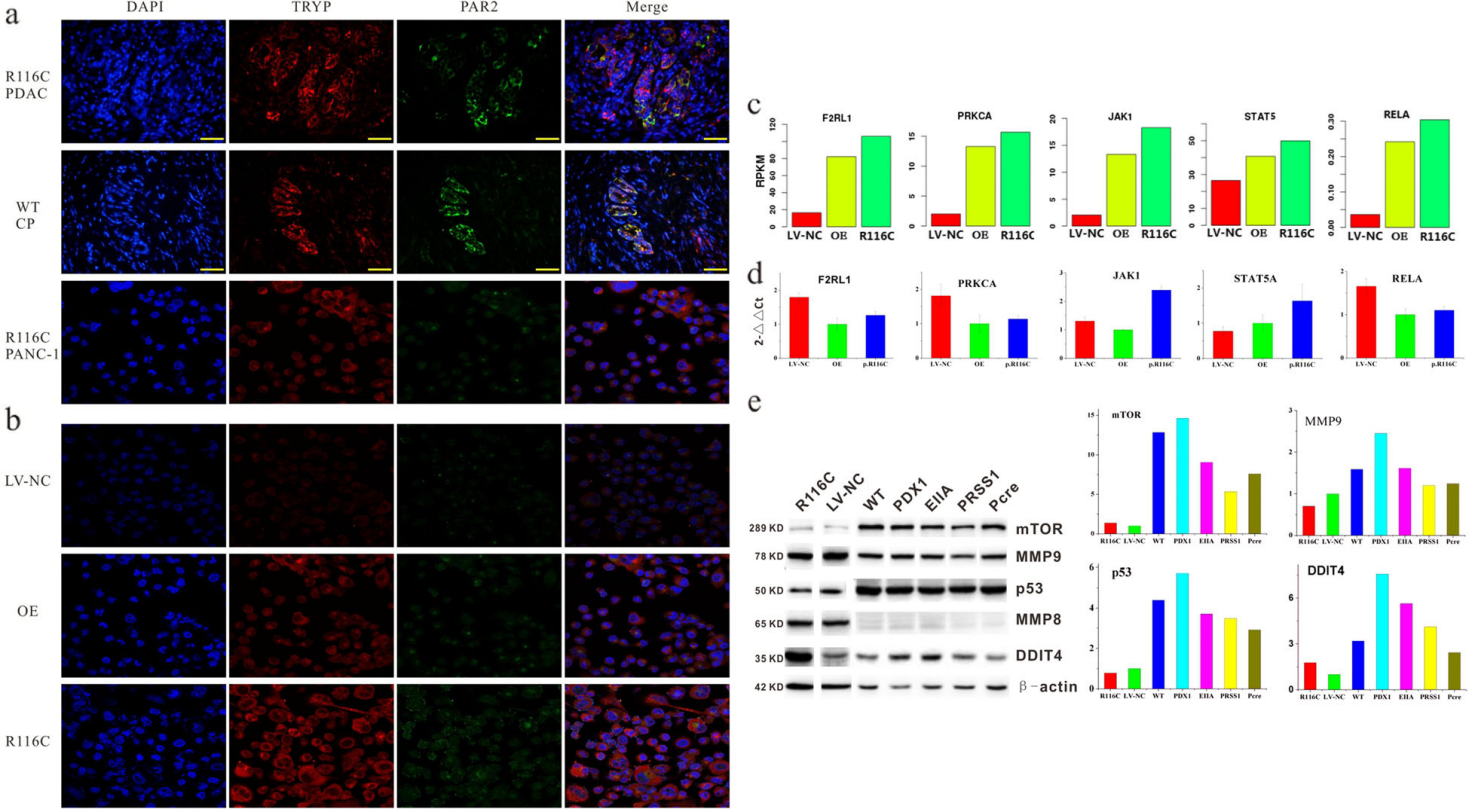
PRSS1_R116C enhances malignant phenotypes in pancreatic cancer via the JAK1/STAT5 pathway. **a**. Trypsin was co-localized with PAR2; **b**. Effect of PRSS1_R116C mutant/PRSS1 wild type overexpression on trypsin and PAR2 expression levels. **c**. RNA-seq was performed to assess the mRNA expression levels of critical proteins in the JAK-STAT pathway; **d**. qRT-PCR was applied to confirm the mRNA levels of critical proteins in the JAK1-STAT5 pathway; **e**. Western blot measurement of tumor relevant proteins

To clarify whether trypsin with the PRSS1_R116C mutation exerts biological effects by binding to receptor PAR-2, similar to trypsin with wild type PRSS1, confocal microscopy was applied to localize mutant-trypsin and PAR-2 proteins in pancreatic cancer tissues and cells. The results showed the two proteins co-localized in both tissues and cells (Fig. 3a). p.R116C mutation leads to an unpaired Cys residue with the potential to interfere with protein folding via incorrect disulfide bond formation. Recombinantly expressed p.R116C trypsinogen exhibited a tendency for misfolding in vitro. The results suggested the R116C mutation maybe induce the expression of trypsin and PAR-2 (Fig. 3b). At the RNA level, RNA-seq (Fig. 3c) and qRT-PCR (Fig. 3d) were performed to assess critical genes in the JAK1-STAT5 pathway. The results showed that F2RL1 (PAR-2) and phosphorylated protein kinase C α (PRKCA), upstream effectors of the JAK1, STAT5, and JAK1/STAT5 canonical pathways, were significantly upregulated in pancreatic cancer cells with the PRSS1_R116C mutation vs OE or LV-NC counterparts; the same expression pattern was applied to REAL (a molecule in the non-canonical JAK1/STAT5 pathway). These findings indicated the PRSS1_R116C mutation not only could promote tumor progression by elevating trypsin expression levels, but also enhanced the malignant behaviors of pancreatic cancer via the JAK1-STAT5 pathway. Subsequently, the expression of tumor relevant markers was detected at the protein level. In cells and the transgenic mouse model, the R116C mutation upregulated mTOR and MMP8/9 (matrix metalloproteinase), with remarkable inductive effects on DNA-damage-inducible transcript 4 (DDIT4), implying that cells harboring PRSS1_R116C have high capability of proliferation and invasion (Fig. 3e).

### PRSS1_R116C mutation promotes carcinogenesis and progression in primary pancreatic cancer

**Fig. 4:**
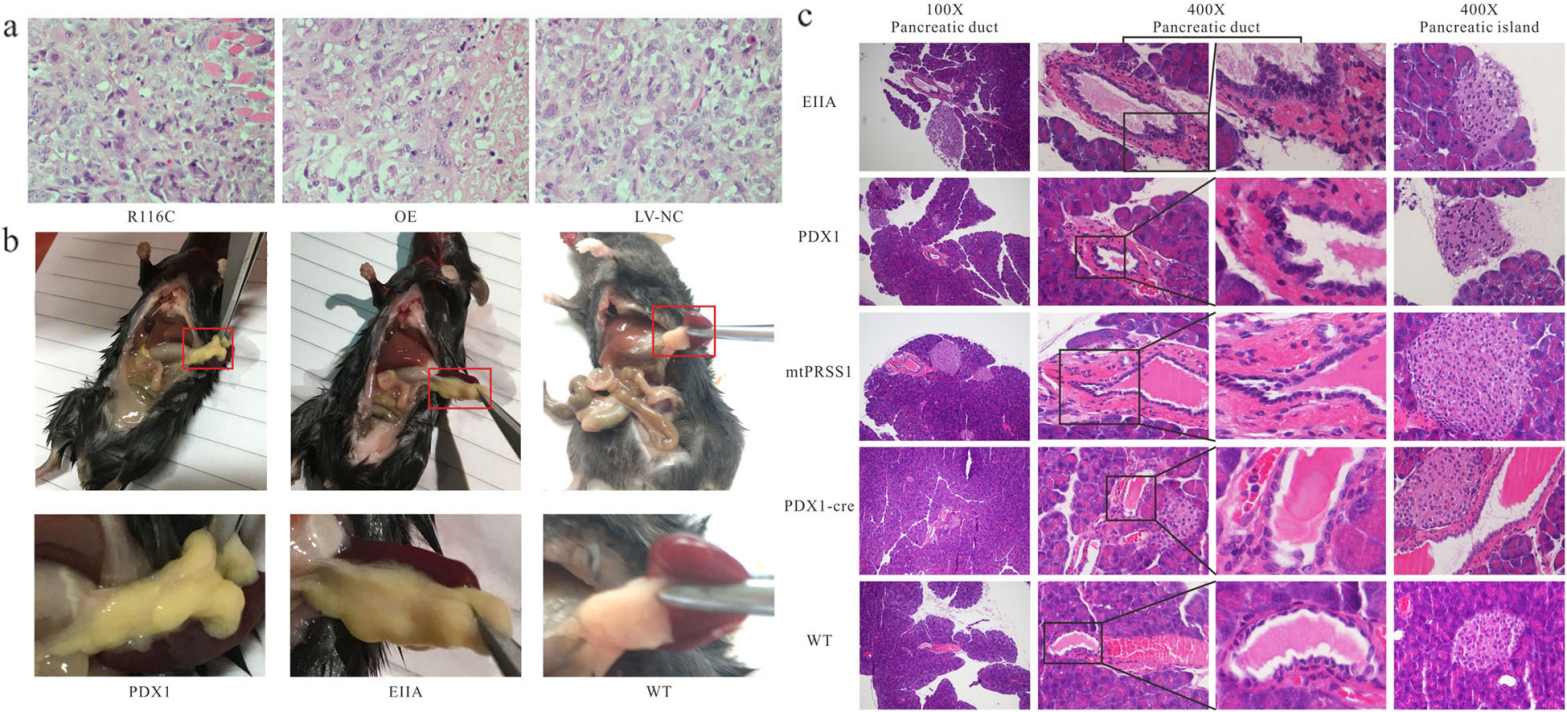
In vivo validation of the inductive effect of R116C mutation on pancreatic carcinogenesis and tumor development. **a**. Tumor-bearing mice, low differentiated adenocarcinoma (400×); **b**. Gross changes in the pancreas of human PRSS1_R116C transgenic mice. **c**. Gross changes in the pancreas of the transgenic mouse model

PANC-1 cells with overexpression of PRSS1_R116C (R116C) or wildtype- PRSS1 (OE) were subcutaneously implanted in nude mice at the same cell number to generate a tumor-bearing mouse model with low differentiation adenocarcinoma. Histopathological examinations showed larger nucleus size, more obvious dyskaryosis, and more cell nucleoli in R116C vs the counterpart cells with OE or LV-NC. The above effects were more pronounced in cells with OE than the LV-NC counterparts. These findings indicated elevated DNA/RNA replication in pancreatic cancer tissues harboring PRSS1_R116C (Fig. 4a). In transgenic mice (70–80 week old), the pancreas showed altered color in PDX1 and EIIA models in comparison with organs from mice with wildtype hPRSS1. Moreover, the pancreas was significantly larger in PRSS1_R116C harboring mice, with a hard texture (Fig. 4b). The pancreas weight showed significant difference between PDX1 (0.4326 ± 0.0606) g and WT (0.2139 ± 0.0415) g (*P* = 0.0023), While there is no significant difference between PDX1 and CIIA (0.3739 ± 0.0338) g (*P* = 0.053). Histopathological examinations revealed pancreatic duct inflammation in all PRSS1_R116C transgenic mice (EIIA and PDX1 lines) (Fig. 4c), reflected by inflammation in the small pancreatic duct with heterogeneity in structure and cell arrangement, disorganization and mucilage of duct epithelial cells, and pancreatic intraepithelial neoplasia (PanINs). In addition, pancreatic islets were present in many regions, appearing like “hyperplasia”.

### Potential mechanism by which R116C mutation promotes pancreatic carcinogenesis and tumor progression

**Fig. 5:**
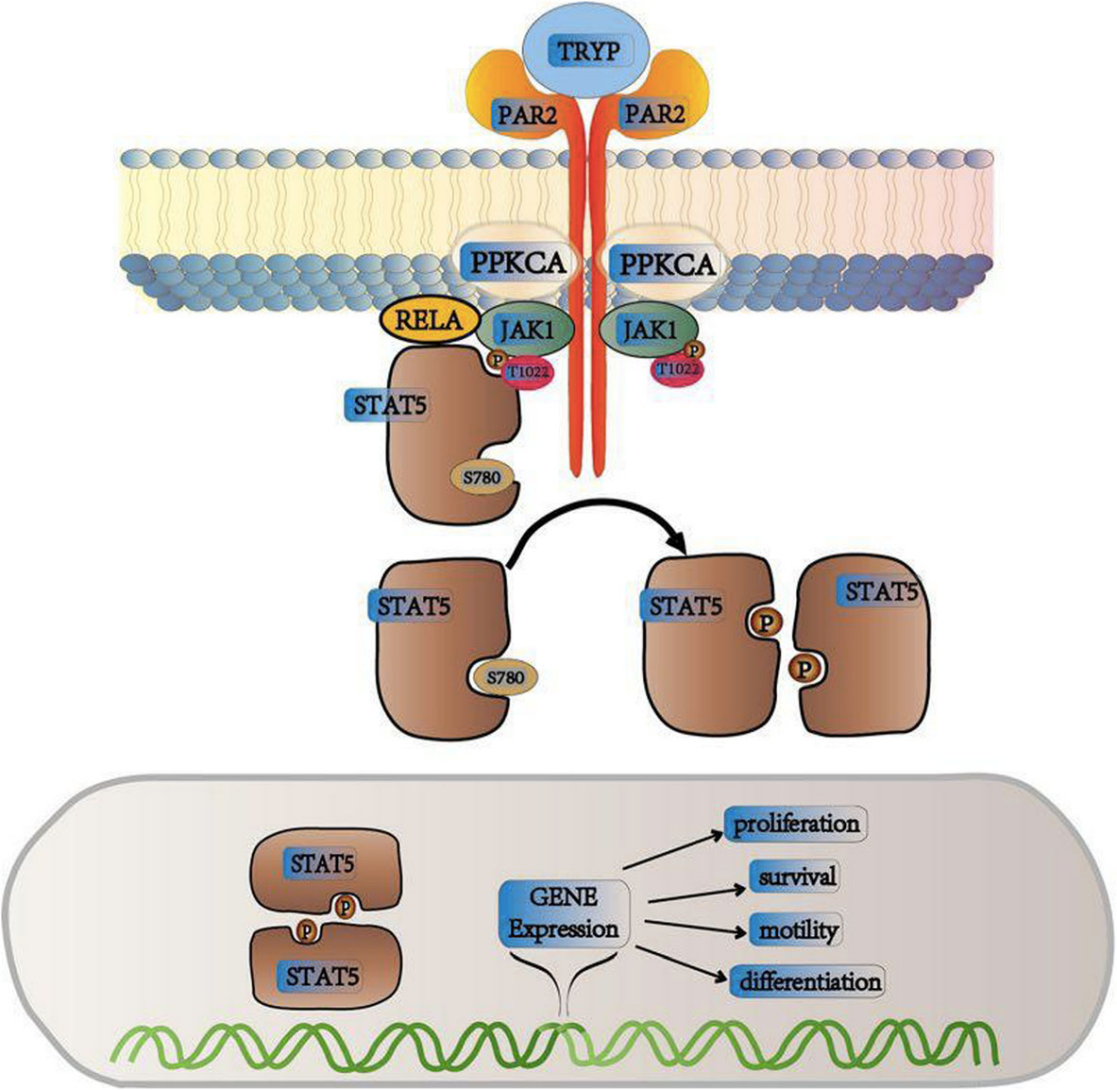
Diagram of the potential mechanism

Binding of trypsin to the native receptor PAR2 causes dimerization of the latter, which brings together receptor-coupled JAK1 kinases, facilitating their mutual activation via cross tyrosine phosphorylation (Fig. 5). Activated JAK1 modulates phosphorylation of tyrosine residues on the receptor, and along with the surrounding amino acids, the phosphorylated tyrosine loci assemble into a docking site, which could be enhanced by PPKCA and RELA (Nuclear Factor NF-Kappa-B P65 Subunit). Meanwhile, STAT5 containing the SH2 domain is recruited to this docking site. Ultimately, kinase JAK1 catalyzes the phosphorylation of receptor-bound STAT5, and activated STAT5 in the polymeric form translocates into the nucleus and binds to target genes, regulating their transcription.

## Discussion

This study uncovered increased serum trypsin levels and PRSS1 mutation in pancreatic cancer patients, and the gene variation was in somatic cells. Because trypsin is specifically and highly expressed in the pancreas, we speculated that trypsin promotes pancreatic carcinogenesis and tumor development in an “inside job” manner. In vitro, exogenous trypsin and trypsin inhibitor addition to serum-free media demonstrated trypsin could indeed promote the proliferation of pancreatic cancer cells. Moreover, PRSS1 mutations of diverse types have been detected in CP and pancreatic cancer in recent years, with clinical and genetic heterogeneity in various mutation types (Liu et al., 2008; McWilliams et al., 2009; Tamura et al., 2018; Németh et al., 2017). In a previous study, it has been shown that protein harboring the p.R116C mutation is poorly secreted compared to wild type and hence induces ER stress (Kereszturi et al., 2009). These findings further confirmed PRSS1 mutation is an important factor in pancreatic cancer. However, it remains unclear whether PRSS1 mutations promote pancreatic carcinogenesis by increasing trypsin expression or directly activating tumor pathways.

The PRSS1_R116C mutation enhanced malignant behaviors in pancreatic cancer by upregulating trypsin. In this study, the PRSS1_R116C mutation rate was relatively high, and serum trypsin levels of these patients were significantly higher than with other mutations. In addition, the PRSS1_R116C mutation was detected in a family at risk for pancreatic cancer as shown above. Therefore, PRSS1_R116C was selected as the functional study object. To assess whether the PRSS1_R116C mutation itself enhances malignant behaviors in pancreatic cancer or triggers the latter by increasing trypsin levels, we constructed stable PANC-1 cell lines with overexpression of PRSS1_R116C (R116C) or wildtype PRSS1 (OE). Cell lysate analysis demonstrated that the PRSS1_R116C mutation resulted in high trypsin expression.

To detect the different effects of PRSS1_R116C mutation and high trypsin levels in promoting pancreatic cancer progression, a series of validation assays were performed. The results demonstrated that the migration rate and invasion capability in pancreatic cancer cells harboring R116C or with OE were significantly increased in comparison with wild type pancreatic cancer cells (LV-NC), with the migration rate in R116C cells also significantly increased compared with OE counterparts. This indicates that trypsin can promote malignant behaviors in pancreatic cancer. However, whether the PRSS1_R116C mutation could elicit primary pancreatic tumor remains unknown. Previous findings demonstrated that 66% of cancer cases are caused by random errors of DNA replication (Tomasetti et al., 2017). In the present study, high trypsin levels resulted in cell cycle arrest in the S phase in tumor cells, without impacting G1/G2 phase transition. Moreover, the PRSS1_R116C mutation itself did not affect the cell cycle, but triggered cell cycle changes by producing high levels of trypsin. The S phase (DNA synthesis phase) is more prone to errors because during its synthesis, DNA is peculiarly sensitive to genotoxic factors and more susceptible to impairment (Tomasetti et al., 2017; Ono et al., 2015; Campbell et al., 2010). This could be the etiology of pancreatic cancer caused by PRSS1 mutation.

The PRSS1_R116C maybe upregulate trypsin and its physiological receptor PAR-2. PAR-2 is a cell surface receptor, present in pancreatic cancer and a variety of other digestive tumors at higher levels than in normal tissues and cells, and closely associated with tumor cell proliferation, invasion and metastasis (Cheng et al., 2017; Piran et al., 2016; Kanemaru et al., 2017; Athwal et al., 2014). RT-qPCR and confocal microscopy demonstrated at the RNA and protein levels, respectively, that PAR-2 was significantly upregulated in pancreatic cancer cells with PRSS1_R116C or with PRSS1 overexpression, and both molecules colocalized in both tissues and cells. These findings indicate both trypsin and its variants activate and upregulate PAR-2, thereby exerting biological effects.

Compared with PANC-1 cells overexpressing wild type PRSS1 (OE), PRSS1_R116C-bearing PANC-1 cells (R116C) had stronger capacity of invasion and migration, indicating that PRSS1_R116C could promote tumor invasion and metastasis via other pathways. After phosphorylation antibody array analysis, RNA-Seq and IPA, followed by validation by qRT-PCR, JAK1/STAT5 signaling was identified as the pathway mediating the effect of PRSS1 mutation. According to previous reports, MMPs are the primary factors mediating tumor invasion and metastasis triggered by trypsin and PAR-2 (Mußbach et al., 2016; Yamashita et al., 2003). In this study, the PRSS1_R116C mutation obviously upregulated MMP8 and MMP9, further confirming the increased invasive and migration abilities of PRSS1_R116C-bearing cells.

PRSS1_R116C enhanced the occurrence of pancreatic duct inflammation and PanINs. In transgenic mouse models with pancreas-specific expression of PRSS1_R116C (PDX1) and systemic production of PRSS1_R116C (EIIA), the pancreas was remarkably enlarged with a hard texture, displaying small pancreatic duct inflammation, with structure and cell heterogeneity, as well as PanINs (Torrente & DeNicola, 2017). In these transgenic mice, the JAK1-STAT5 pathway was activated in the pancreas. In vitro and in vivo experiments, particularly the transgenic mouse models, provided direct evidence that R116C can trigger pancreatic cancer or precancerous changes.

## Conclusions

We uncovered a novel pathway mediating pancreatic cancer development. PRSS1 mutation and overexpression plays an “inside job” role in pancreatic carcinogenesis and tumor development. These findings could help address clinical questions regarding the corresponding disease from a better perspective; for example they explain, at least in part, why pancreatic cancer is so aggressive and progresses so rapidly. Moreover, we established transgenic mouse models to successfully confirm that PRSS1_R116C mutation may induce primary pancreatic cancer. Meanwhile, the study suggests that anti-trypsin intervention could become a new tool for inhibiting pancreatic carcinogenesis and tumor development.

## Supplementary information

**Additional file 1:** Two families were recruited for whole-exome sequencing screening. Family a. 2 cases of PDAC (I1, II3) and 3 healthy first-degree relatives (I2, II2, and II5); family b. 1 case of PDAC (II3), 1 case of chronic pancreatitis (I1), and 2 healthy first-degree relatives (I2, II2). (DOCX 72 kb)

**Additional file 2:** High trypsin level is a risk factor for pancreatic cancer. a. Serum trypsin levels were significantly higher in pancreatic cancer patients than in healthy controls, but lower than in pancreatitis patients; b. Trypsin intervention experiment (CCK8 assay); Three experiments at different times, each doing three biological duplication; c. Inhibitory effect of trypsin inhibitor on tumor cell growth; Three experiments at different times, each doing three biological duplication; d. Pathological features of tumor microenvironment and Treg cell distribution in CP and PDAC; e. Distribution of pancreatic duct epithelial cells in PDAC and CP. (DOCX 217 kb)

**Additional file 3:** Ingenuity Pathway Analysis (IPA) of phosphorylation antibody array data was performed to identify the pathways impacted by the PRSS1_R116C mutation. (DOCX 161 kb)

**Additional file 4:** Phosphorylated proteins with differential expression between R116C and LV-NC. (DOCX 17 kb)

**Additional file 5:** RNA-Seq screening for differential mRNA expression of genes impacted by the R116C mutation. (DOCX 83 kb)

**Additional file 6:** Transgenic mice were used to validate the potential pathway which likely mediated R116C mutation-associated induction of pancreatic carcinogenesis. With pancreatic tissue samples from transgenic mice as study object, qRT-PCR was performed to validate the pathway involved in R116C mutation-associated induction of pancreatic pathogenesis. (DOCX 69 kb)

## Data Availability

All data and materials generated during and/or analysed during the current study are available from the corresponding author on reasonable request.

## Abbreviations

DAPI: Diamidino-2-phenylindole
ECM: Extracellular matrix
EIIA: Systemic hPRSS1_R116C mutant transgenic mouse produced by breeding of the founder mouse with EIIA-Cre heterozygote
EIIA-Cre: Cre recombinase under the EIIA promoter
ELISA: Enzyme-linked immunosorbent analyses
ERK: Extracellular signal-regulated kinase
FITC: Fluorescein isothiocyanate
mtPRSS1: transgenic mouse without activation before breeding to a Cre harboring mouse
OE: Overexpressing
PanINs: Pancreatic intraepithelial neoplasia
PAR-2: Protease-activated receptor-2
PDAC: Pancreatic ductal adenocarcinoma
PDX1: pancreas specific hPRSS1_R116C mutant transgenic mouse produced by breeding of the founder mouse with a Pdx1-Cre heterozygote
Pdx1-Cre: SJ-014647, mouse Pdx1 (pancreatic and duodenal homeobox 1); Cre activity could be detected in the pancreatic duct epithelium, gastric antrum, duodenal antrum, and pancreatic islet
PRSS1: Cationic trypsinogen gene
RNA-seq: Transcriptome deep sequencing
SNPs: Single nucleotide polymorphisms

## References

[CR1] Athwal T, Huang W, Mukherjee R, Latawiec D, Chvanov M, Clarke R, Smith K, Campbell F, Merriman C, Criddle D (2014). Expression of human cationic trypsinogen (PRSS1) in murine acinar cells promotes pancreatitis and apoptotic cell death. Cell Death Dis.

[CR2] Campa D, Pastore M, Capurso G, Hackert T, Di Leo M, Izbicki JR, Khaw KT, Gioffreda D, Kupcinskas J, Pasquali C (2018). Do pancreatic cancer and chronic pancreatitis share the same genetic risk factors? A PANcreatic disease ReseArch (PANDoRA) consortium investigation. Int J Cancer.

[CR3] Campbell PJ, Yachida S, Mudie LJ, Stephens PJ, Pleasance ED, Stebbings LA, Morsberger LA, Latimer C, McLaren S, Lin ML (2010). The patterns and dynamics of genomic instability in metastatic pancreatic cancer. Nature..

[CR4] Chang MC, Wu CH, Yang SH, Liang PC, Chen BB, Jan IS, Chang YT, Jeng YM (2017). Pancreatic cancer screening in different risk individuals with family history of pancreatic cancer-a prospective cohort study in Taiwan. Am J Cancer Res.

[CR5] Cheng RKY, Fiez-Vandal C, Schlenker O, Edman K, Aggeler B, Brown DG, Brown GA, Cooke RM, Dumelin CE, Doré AS (2017). Structural insight into allosteric modulation of protease-activated receptor 2. Nature..

[CR6] Hezel AF, Kimmelman AC, Stanger BZ, Bardeesy N, Depinho RA (2006). Genetics and biology of pancreatic ductal adenocarcinoma. Genes Dev.

[CR7] Jiang G, Cao F, Ren G, Gao D, Bhakta V, Zhang Y, Cao H, Dong Z, Zang W, Zhang S (2010). PRSS3 promotes tumour growth and metastasis of human pancreatic cancer. Gut..

[CR8] Kanemaru A, Yamamoto K, Kawaguchi M, Fukushima T, Lin CY, Johnson MD, Camerer E, Kataoka H (2017). Deregulated matriptase activity in oral squamous cell carcinoma promotes the infiltration of cancer-associated fibroblasts by paracrine activation of protease-activated receptor 2. Int J Cancer.

[CR9] Kereszturi E, Szmola R, Kukor Z, Simon P, Weiss FU, Lerch MM, Sahin-Tóth M (2009). Hereditary pancreatitis caused by mutation-induced misfolding of human cationic trypsinogen: a novel disease mechanism. Hum Mutat.

[CR10] Kleeff J, Whitcomb DC, Shimosegawa T, Esposito I, Lerch MM, Gress T, Mayerle J, Drewes AM, Rebours V, Akisik F (2017). Chronic pancreatitis. Nat Rev Dis Primers.

[CR11] Le Maréchal C, Masson E, Chen JM, Morel F, Ruszniewski P, Levy P, Férec C (2006). Hereditary pancreatitis caused by triplication of the trypsinogen locus. Nat Genet.

[CR12] Liu Q, Lin X, Liu J, Liu A, Gao F (2012). The −409 C/T genotype of PRSS1 protects against pancreatic cancer in the Han Chinese population. Dig Dis Sci.

[CR13] Liu QC, Gao F, Ou QS, Zhuang ZH, Lin SR, Yang B, Cheng ZJ (2008). Novel mutation and polymorphism of PRSS1 gene in the Chinese patients with hereditary pancreatitis and chronic pancreatitis. Chin Med J.

[CR14] Lowenfels AB, Maisonneuve P, Cavallini G, Ammann RW, Lankisch PG, Andersen JR, Dimagno EP, Andrén-Sandberg A, Domellöf L (1993). Pancreatitis and the risk of pancreatic cancer. International Pancreatitis Study Group. N Engl J Med.

[CR15] McWilliams RR, Bamlet WR, de Andrade M, Rider DN, Couch FJ, Cunningham JM, Matsumoto ME, Rabe KG, Hammer TJ, Petersen GM (2009). Polymorphic variants in hereditary pancreatic cancer genes are not associated with pancreatic cancer risk. Cancer Epidemiol Biomark Prev.

[CR16] Mußbach F, Ungefroren H, Günther B, Katenkamp K, Henklein P, Westermann M, Settmacher U, Lenk L, Sebens S, Müller JP (2016). Proteinase-activated receptor 2 (PAR2) in hepatic stellate cells-evidence for a role in hepatocellular carcinoma growth in vivo. Mol Cancer.

[CR17] Németh BC, Patai ÁV, Sahin-Tóth M, Hegyi P (2017). Misfolding cationic trypsinogen variant p.L104P causes hereditary pancreatitis. Gut..

[CR18] Ono H, Basson MD, Ito H (2015). PTK6 potentiates gemcitabine-induced apoptosis by prolonging S-phase and enhancing DNA damage in pancreatic Cancer. Mol Cancer Res.

[CR19] Piran R, Lee SH, Kuss P, Hao E, Newlin R, Millán JL, Levine F (2016). PAR2 regulates regeneration, transdifferentiation, and death. Cell Death Dis.

[CR20] Rebours V, Boutron-Ruault MC, Schnee M, Férec C, Maire F, Hammel P, Ruszniewski P, Lévy P (2008). Risk of pancreatic adenocarcinoma in patients with hereditary pancreatitis: a national exhaustive series. Am J Gastroenterol.

[CR21] Rustgi AK (2014). Familial pancreatic cancer: genetic advances. Genes Dev.

[CR22] Soreide K, Janssen EA, Körner H, Baak JP (2006). Trypsin in colorectal cancer: molecular biological mechanisms of proliferation, invasion, and metastasis. J Pathol.

[CR23] Tamura K, Yu J, Hata T, Suenaga M, Shindo K, Abe T, MacGregor-Das A, Borges M, Wolfgang CL, Weiss MJ (2018). Mutations in the pancreatic secretory enzymes CPA1 and CPB1 are associated with pancreatic cancer. Proc Natl Acad Sci U S A.

[CR24] Tomasetti C, Li L, Vogelstein B (2017). Stem cell divisions, somatic mutations, cancer etiology, and cancer prevention. Science..

[CR25] Torrente L, DeNicola GM (2017). Stressing out PanIN: NRF2 pushes over the edge. Cancer Cell.

[CR26] Yamashita K, Mimori K, Inoue H, Mori M, Sidransky D (2003). A tumor-suppressive role for trypsin in human cancer progression. Cancer Res.

