# Supplementary material for: PRSS1 mutation: a possible pathomechanism of pancreatic carcinogenesis and pancreatic cancer
Source: Mol Med. 2019 Sep 14;25:44. doi: 10.1186/s10020-019-0111-4 (PMC6744682; doi:10.1186/s10020-019-0111-4)
Supplement: Supplementary file 1 — Additional file 1: Two families were recruited for whole-exome sequencing screening. Family a. 2 cases of PDAC (I1, II3) and 3 healthy first-degree relatives (I2, II2, and II5); family b. 1 case of PDAC (II3), 1 case of chronic pancreatitis (I1), and 2 healthy first-degree relatives (I2, II2). (DOCX 72 kb) [file 10020_2019_111_MOESM1_ESM.docx]

**Additional file 1. Two families were recruited for whole-exome sequencing screening**

Family a. 2 cases of PDAC (I1, II3) and 3 healthy first-degree relatives (I2, II2, and II5); family b. 1 case of PDAC (II3), 1 case of chronic pancreatitis (I1), and 2 healthy first-degree relatives (I2, II2)

**
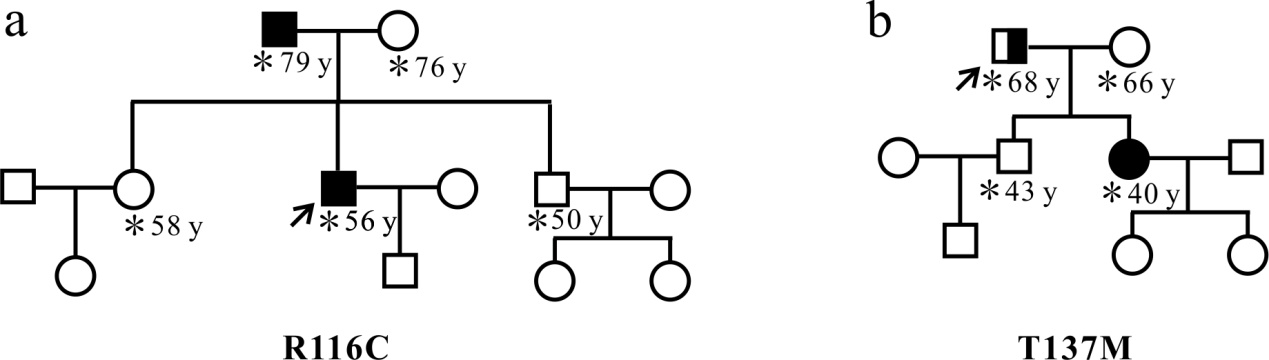
**
