# Supplementary material for: PRSS1 mutation: a possible pathomechanism of pancreatic carcinogenesis and pancreatic cancer
Source: Mol Med. 2019 Sep 14;25:44. doi: 10.1186/s10020-019-0111-4 (PMC6744682; doi:10.1186/s10020-019-0111-4)

**Additional file 2. High trypsin level is a risk factor for pancreatic cancer.**

a. Serum trypsin levels were significantly higher in pancreatic cancer patients than in healthy controls, but lower than in pancreatitis patients; b. Trypsin intervention experiment (CCK8 assay); Three experiments at different times, each doing three biological duplication; c. Inhibitory effect of trypsin inhibitor on tumor cell growth; Three experiments at different times, each doing three biological duplication; d. Pathological features of tumor microenvironment and Treg cell distribution in CP and PDAC; e. Distribution of pancreatic duct epithelial cells in PDAC and CP.


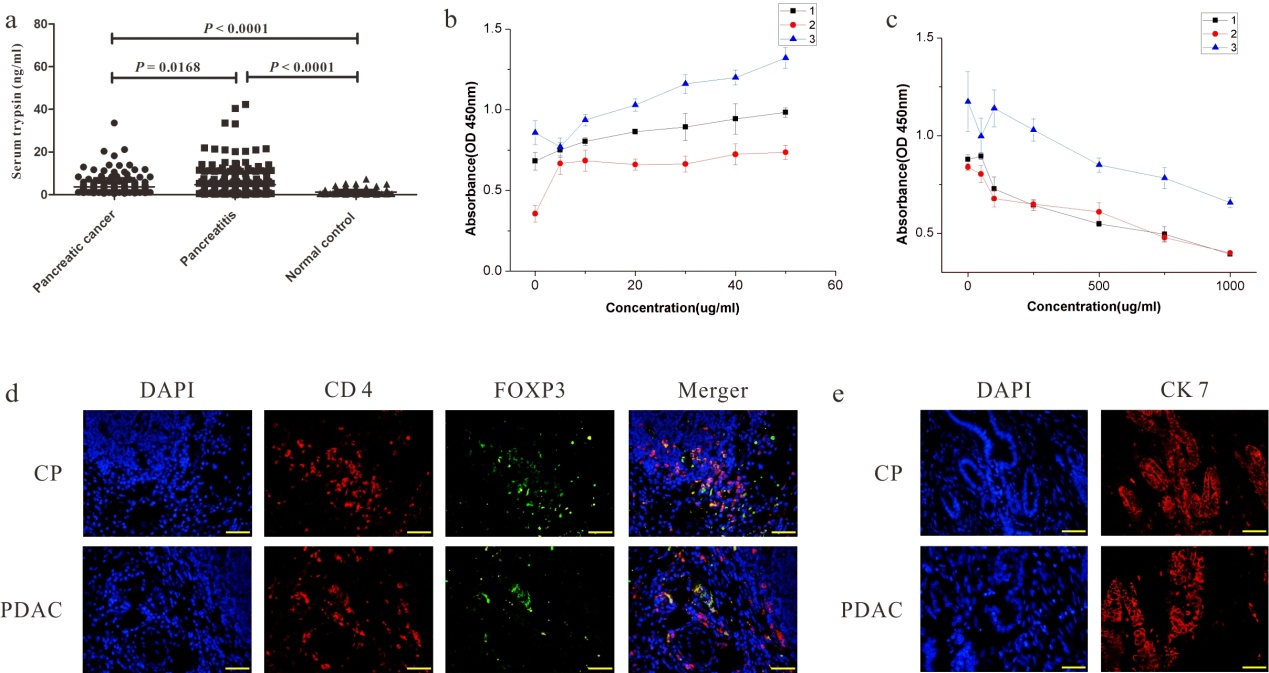

Supplement: Supplementary file 2 — Additional file 2: High trypsin level is a risk factor for pancreatic cancer. a. Serum trypsin levels were significantly higher in pancreatic cancer patients than in healthy controls, but lower than in pancreatitis patients; b. Trypsin intervention experiment (CCK8 assay); Three experiments at different times, each doing three biological duplication; c. Inhibitory effect of trypsin inhibitor on tumor cell growth; Three experiments at different times, each doing three biological duplication; d. Pathological features of tumor microenvironment and Treg cell distribution in CP and PDAC; e. Distribution of pancreatic duct epithelial cells in PDAC and CP. (DOCX 217 kb) [file 10020_2019_111_MOESM2_ESM.docx]
