# Supplementary material for: PRSS1 mutation: a possible pathomechanism of pancreatic carcinogenesis and pancreatic cancer
Source: Mol Med. 2019 Sep 14;25:44. doi: 10.1186/s10020-019-0111-4 (PMC6744682; doi:10.1186/s10020-019-0111-4)

**Additional file 3. Ingenuity Pathway Analysis (IPA) of phosphorylation antibody array data was performed to identify the pathways impacted by the PRSS1_R116C mutation.**


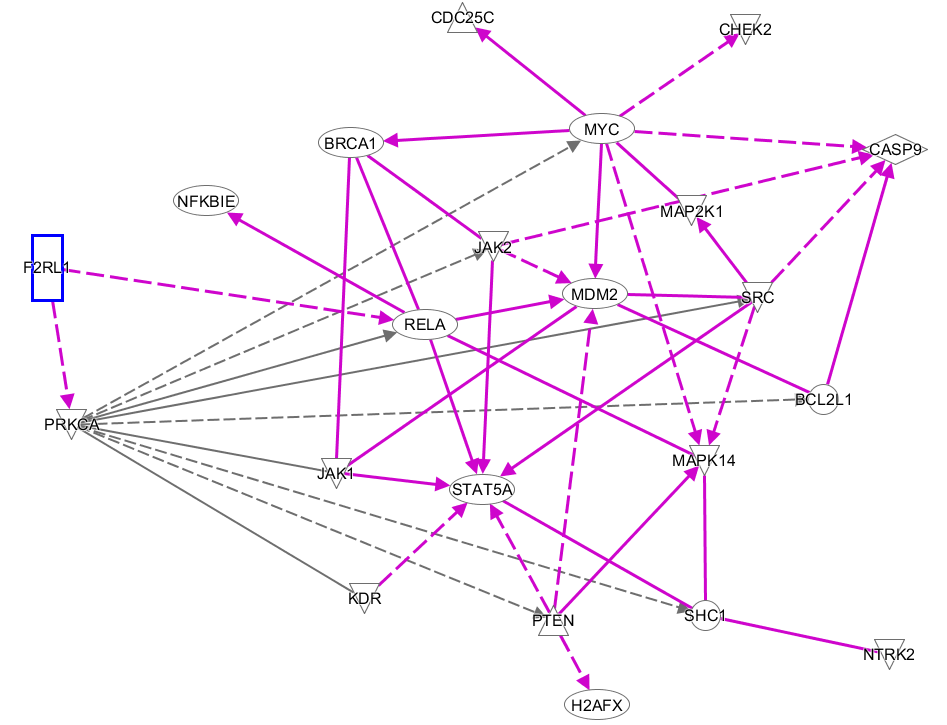

Supplement: Supplementary file 3 — Additional file 3: Ingenuity Pathway Analysis (IPA) of phosphorylation antibody array data was performed to identify the pathways impacted by the PRSS1_R116C mutation. (DOCX 161 kb) [file 10020_2019_111_MOESM3_ESM.docx]
