# Supplementary material for: PRSS1 mutation: a possible pathomechanism of pancreatic carcinogenesis and pancreatic cancer
Source: Mol Med. 2019 Sep 14;25:44. doi: 10.1186/s10020-019-0111-4 (PMC6744682; doi:10.1186/s10020-019-0111-4)
Supplement: Supplementary file 4 — Additional file 4: Phosphorylated proteins with differential expression between R116C and LV-NC. (DOCX 17 kb) [file 10020_2019_111_MOESM4_ESM.docx]

**Additional file 4.** phosphorylated proteins with differential expression between R116C and LV-NC

| Name | Gene Syml | Swiss Prot | LV-NC_-_Phos/Unphos | 900_-_Phos/Unphos | **FC≧1.2_-_900_-_VS_-_LV_-_NC** |
| --- | --- | --- | --- | --- | --- |
| BRCAl (Phospho-Ser1423) | BRCAl | P38398 | 0.12 | 0.23 | 1.91 |
| BCL-XL (Phospho-Ser62) | BCL2L1 | Q07817 | 0.09 | 0.15 | 1.67 |
| MDM2 (Phospho-Ser166) | MDM2 | Q00987 | 0.16 | 0.27 | 1.64 |
| STAT6 (Phospho-Thr645) | STAT6 | P42226 | 0.19 | 0.28 | 1.46 |
| Caspase 9 (Phospho-Ser196) | CASP9 | P55211 | 0.35 | 0.50 | 1.45 |
| VEGFR2 (Phospho-Tyr951) | KDR | P35968 | 0.15 | 0.21 | 1.45 |
| STATSA (Phospho-Ser780) | STATSA | P42229 | 0.05 | 0.07 | 1.35 |
| Shc (Phospho-Tyr349) | SHC 1 | P29353 | 0.20 | 0.27 | 1.34 |
| Myc (Phospho-Thr358) | MYC | P01106 | 0.11 | 0.14 | 1.30 |
| Trk B (Phospho-Tyr515) | NTRK2 | Q16620 | 0.13 | 0.16 | 1.28 |
| MEKl (Phospho-Thr291) | MAP2K1 | Q02750 | 0.31 | 0.39 | 1.26 |
| Chk2 (Phospho-Thr68) | CHEK2 | O96017 | 0.21 | 0.26 | 1.25 |
| cdc25C (Phos-pho-Ser216) | CDC25C | P30307 | 0.42 | 0.52 | 1.25 |
| Src (Phospho-Tyr418) | SRC | P12931 | 0.17 | 0.21 | 1.25 |
| BRCAl (Phospho-Ser1524) | BRCAl | P38398 | 0.49 | 0.59 | 1.22 |
| Caspase 9 (Phospho-Tyr153) | CASP9 | P55211 | 1.08 | 1.31 | 1.21 |
| JAKl (Phospho-Tyr1022) | JAKl | P23458 | 0.67 | 0.80 | 1.20 |
| PTEN (Phospho-Ser380/Thr382/Thr3 | PTEN | P60484 | 0.54 | 0.65 | 1.20 |
| lkB-epsilon (Phospho-Ser22) | NFKBIE | O00221 | 0.19 | 0.21 | 1.13 |
| P38 MAPK (Phospho-Tyr182) | MAPK14 | Q16539 | 0.33 | 0.37 | 1.13 |
| NFkB-plOO/p52 (Phospho-Ser865) | RELA | Q04206 | 0.44 | 0.50 | 1.13 |
| Histone H2AX (Phospho-Ser139) | H2AFX | P16104 | 0.58 | 0.65 | 1.12 |
| JAK2 (Phospho-Tyr1007) | JAK2 | O60674 | 0.50 | 0.56 | 1.12 |
| p27Kipl (Phospho-Thr187) | CDKN1B | P46527 | 0.50 | 0.44 | 0.89 |
| eIF4E (Phospho-Ser209) | EIF4E | P06730 | 0.64 | 0.57 | 0.88 |
| HSFl (Phospho-Ser303) | HSFl | Q00613 | 0.56 | 0.50 | 0.88 |
| BAD (Phospho-Ser155) | BAD | Q92934 | 0.64 | 0.56 | 0.87 |
| P53 (Phospho-Ser315) | TP53 | P04637 | 0.12 | 0.10 | 0.86 |
| Estrogen Receptor-alpha (Phospho-Ser) | ESR1 | P03372 | 0.60 | 0.51 | 0.85 |
| NFkB-p105/p50 (Phospho-Ser907) | NFKB1 | P19838 | 0.73 | 0.62 | 0.85 |
| GSK3 beta (Phospho-Ser9) | GSK3B | P49841 | 1.41 | 1.19 | 0.85 |
| c-Kit (Phospho-Tyr721) | KIT | P10721 | 0.46 | 0.38 | 0.84 |
| NFkB-p65 (Phospho-Thr254) | RELA | Q04206 | 0.73 | 0.61 | 0.84 |
| c-Jun (Phospho-Ser73) | JUN | P05412 | 0.11 | 0.08 | 0.75 |
| lkB-alpha (Phospho-Ser32/Ser36) | NFKBIA | P25963 | 6.88 | 4.19 | 0.61 |
