# Supplementary material for: PRSS1 mutation: a possible pathomechanism of pancreatic carcinogenesis and pancreatic cancer
Source: Mol Med. 2019 Sep 14;25:44. doi: 10.1186/s10020-019-0111-4 (PMC6744682; doi:10.1186/s10020-019-0111-4)
Supplement: Supplementary file 5 — Additional file 5: RNA-Seq screening for differential mRNA expression of genes impacted by the R116C mutation. (DOCX 83 kb) [file 10020_2019_111_MOESM5_ESM.docx]

**Additional file 5.** RNA-Seq screening for differential mRNA expression of genes impacted by the R116C mutation.

**
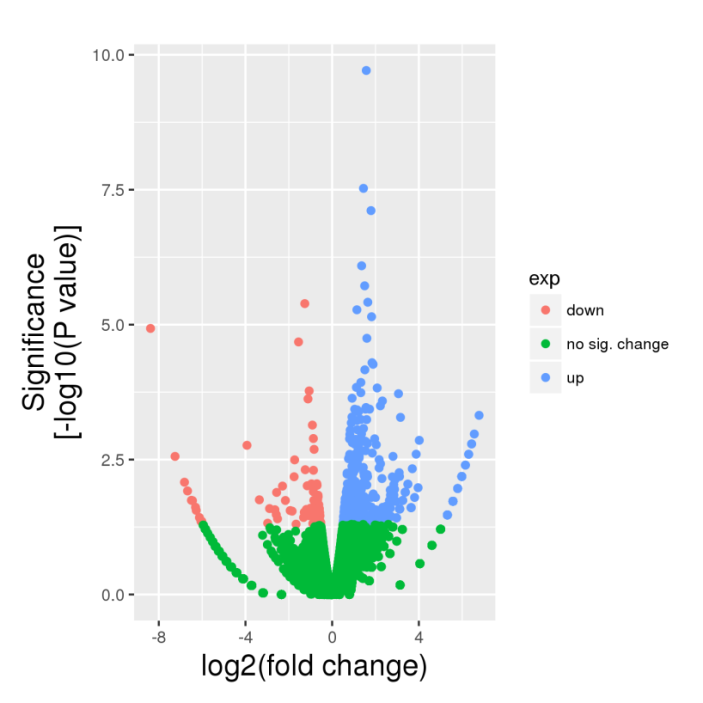
**
