# Supplementary material for: PRSS1 mutation: a possible pathomechanism of pancreatic carcinogenesis and pancreatic cancer
Source: Mol Med. 2019 Sep 14;25:44. doi: 10.1186/s10020-019-0111-4 (PMC6744682; doi:10.1186/s10020-019-0111-4)
Supplement: Supplementary file 6 — Additional file 6: Transgenic mice were used to validate the potential pathway which likely mediated R116C mutation-associated induction of pancreatic carcinogenesis. With pancreatic tissue samples from transgenic mice as study object, qRT-PCR was performed to validate the pathway involved in R116C mutation-associated induction of pancreatic pathogenesis. (DOCX 69 kb) [file 10020_2019_111_MOESM6_ESM.docx]

**Additional file 6.** Transgenic mice were used to validate the potential pathway which likely mediated R116C mutation-associated induction of pancreatic carcinogenesis. With pancreatic tissue samples from transgenic mice as study object, qRT-PCR was performed to validate the pathway involved in R116C mutation-associated induction of pancreatic pathogenesis.

**
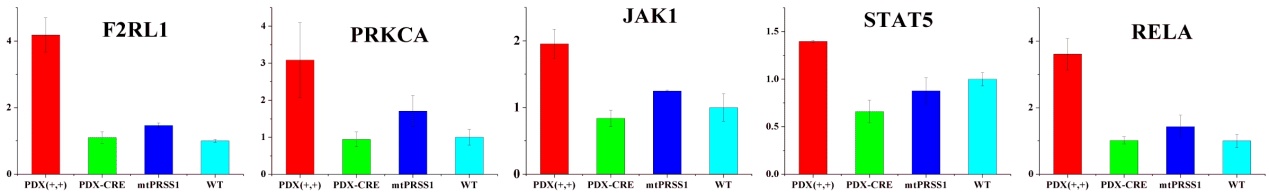
**
